# Virtual Planning and 3D printing modeling for mandibular reconstruction with fibula free flap

**DOI:** 10.4317/medoral.22295

**Published:** 2018-04-24

**Authors:** Wenhao Ren, Ling Gao, Shaoming Li, Cheng Chen, Fan Li, Qibo Wang, Yuan Zhi, Jianzhong Song, Zhichao Dou, Lingfa Xue, Keqian Zhi

**Affiliations:** 1 Department of oral and maxillofacial surgery, The affiliated hospital of Qingdao University, Qingdao, Shandong, P. R. China; 2 Department of oral and maxillofacial surgery, College of Stomatology, Xi’an Jiaotong University, Xi’an, Shaanxi, P. R. China; 3 Department of General Dentistry, College of Stomatology, Xi’an Jiaotong University, Xi’an, Shaanxi, P. R. China; 4 Xiangya School of Stomatology, Central South University, Changsha, Hunan,P.R.China

## Abstract

**Background:**

This study was to evaluate the use of virtual planning and 3D printing modeling in mandibular reconstruction and compare the operation time and surgical outcome of this technique with conventional method.

**Material and Methods:**

Between 2014 and 2017, 15 patients underwent vascularized fibula flap mandibular reconstruction using virtual planning and 3D printing modeling. Titanium plates were pre-bent using the models and cutting guides were used for osteotomies. 15 patients who underwent mandibular reconstruction using fibula flap without aid of virtual planning and 3D printing models were selected as control group. The operation time was recorded and compared in two groups. Accuracy of reconstruction was measured by superimposing the preoperative image onto the postoperative image of mandible. The selected bony landmark, distance and angle were measured.

**Results:**

The mean total operation time and reconstruction time were 1.60±0.37 and 5.54±0.50 hours in computer-assisted group, respectively; These were 2.58±0.45 and 6.54±0.70 hours in conventional group, respectively. Both operation time and reconstruction time were shorter in computer-assisted group. The difference between the preoperative and postoperative intercondylar distances, intergonial angle distances, anteroposterior distances and gonial angles were 2.92±1.15 and 4.48±1.41mm, 2.93±1.19 and 4.79±1.48mm, 4.31±1.24 and 5.61±1.41mm, 3.85±1.68° and 5.88±2.12° in the computer-assisted and conventional group, respectively. The differences between the preoperative and postoperative mandible is smaller in the computer-assisted group.

**Conclusions:**

Virtual planning and 3D printing modeling have the potential to increase mandibular reconstruction accuracy and reduce operation time. we believe that this technology for mandibular reconstruction in selected patients will become a used method and improve the quality of reconstruction.

** Key words:**Mandibular reconstruction, fibula flap, virtual planning, computer-assisted design, 3D printing.

## Introduction

Mandibular reconstruction after ablative tumour removal is still a challenging task to head and neck surgeons, which aims to achieve the best possible functional and esthetic outcomes. Hidalgo reported the utility of vascularized fibula flaps for mandibular reconstruction in 1989 (1), Since then, the fibular free flap has become the first option for mandible reconstruction (2,3). This flap has many advantages, including high quality of long bicortical bone grafts, long pedicle, wide vessel, and the ability to incorporate skin and muscle which are required for mandibular reconstruction (4-6). However, the mobility of the mandible increases the difficulty in the appropriate position of fibula flap to achieve the ideal functional and esthetic outcomes.

The sharping and position of fibula free flap in mandibular reconstruction was based on the surgeon’s experience in the past. This operation is difficult to control during conventional surgery and occasionally result in dissatisfying occlusion and appearance. Now, the virtual planning and three-dimensional (3D) printing modeling using preoperative computed tomographic (CT) data has been introduced to permit more accurate reconstruction (7-9). Base on the data, we can simulate the resection of mandibular bone, segment and shaping of fibular flap and transfer the virtual plan to intraoperative templates. The true-to-size models and templates is easy to obtain by the 3D printing technology. Pre-bending of titanium plate was allowed on the model. These techniques help the surgeons to achieve near perfect position of the pieces of fibular flap.

Several studies have reported that the virtual surgical planning could help to reduce operative time and increase accuracy in mandibular reconstruction with fibula free flap (8,10-12). In this study, we describe our protocol of the mandibular reconstruction with fibula flap using virtual planning and 3D printing techniques. To evaluate the use of virtual planning and 3D printing modeling to improve the accuracy and speed of mandibular reconstruction. We reviewed our experience and compare outcomes in patients who underwent mandibular reconstruction with the aid of this technique to outcomes in patients with conventional method.

## Material and Methods

-Patients

We retrospectively reviewed the records of 15 patients who had undergone mandibulectomy and mandibular reconstruction with fibula free flaps using Virtual planning and 3D printing modeling between July 2014 and June 2017 at Department of Oral and Maxillofacial Surgery, the affiliated hospital of Qingdao University and College of Stomatology Xi’an Jiaotong University. The inclusion criteria were 1) stable occlusal status; 2) division of the free fibula into 2 or more segments. 15 patients who underwent mandibular reconstruction with the conventional method were selected as control group (conventional group). The defects of patients in two group were matched. All tumor resections and mandibular reconstructions were performed by the same team. The Ethics Committee of the College of Medicine proved the study. All patients gave written consent to their inclusion in the study.

-Techniques

**Figure 1:**
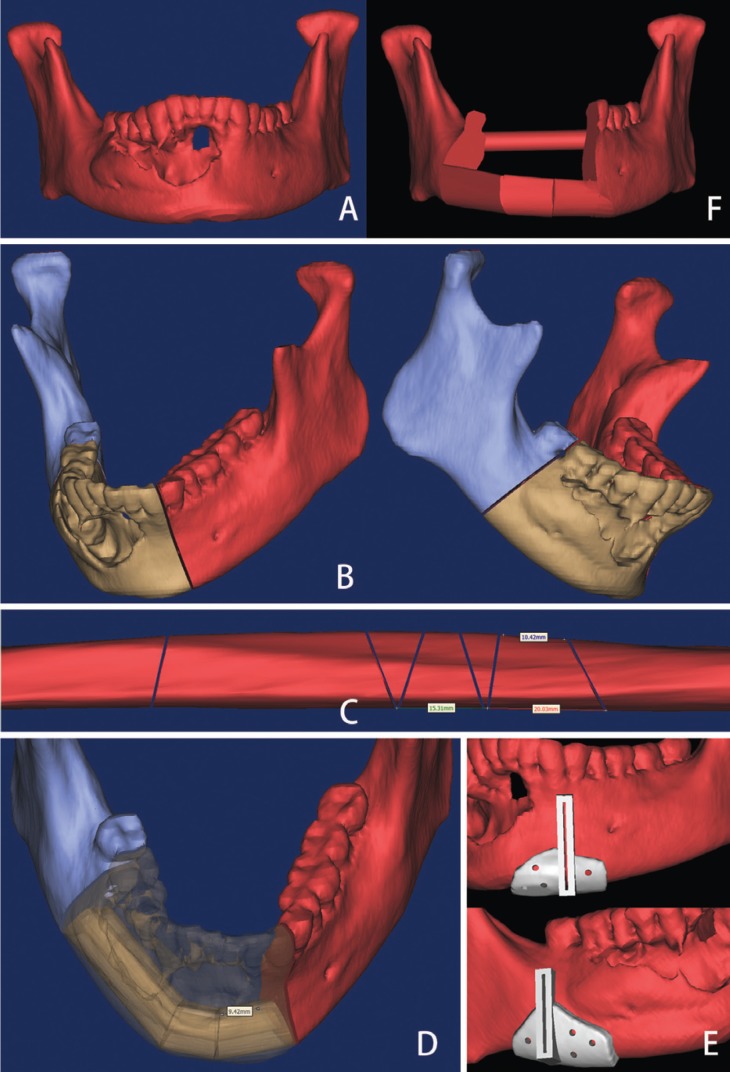
Computer assisted design and virtual planning (A. 3D virtual models of the maxillofacial skeleton; B. Simulation of mandibular osteotomies; C. Simulation of fibular osteotomies; D. Shaping and placement of fibular bone; E. Cutting guide design; F. Virtual mandible reconstruction using fibular bone).

The process of virtual planning began with high-resolution axial computed tomography (CT) scans using fine-cut (0.45 mm) of maxillofacial skeleton and lower extremities. Images were saved in DICOM (Digital Imaging and Communications in Medicine) format and imported to the Mimics 16.0 (Materialise, Leuven, Belgium). 3D virtual models of the maxillofacial skeleton and fibula, as well as the simulation of mandibular osteotomies was performed using this software (Fig. 1). In the process of virtual mandibular resection and fibular osteotomies, the designer work with the surgeon and confirm the osteotomies line together. The shaping and placement of fibular bone were planned by visualizing the reconstruction superimposed on the preoperative image of the mandible such that the outer (inferior-lateral mandibular border) contour of the mandible was restored. If the contour of the mandible was destroyed by the tumor, mirroring tools were used.

**Figure 2:**
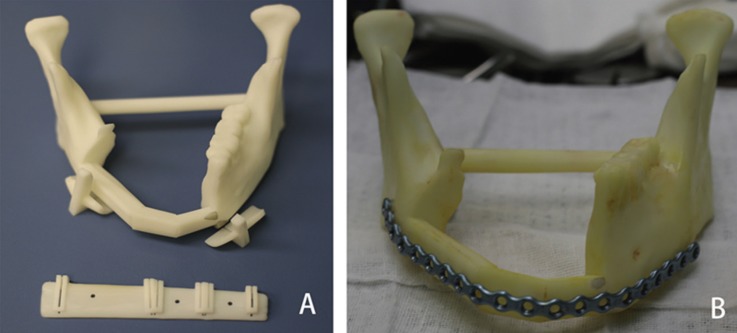
3D printing modeling (A. The mandibular model and cutting guide was manufactured by the 3D printer; B. The titanium reconstruction plate was pre-bent along the contours of the model).

When the virtual surgery was finished, design of the cutting guide was beginning which allow the surgeon to precisely resect the lesion of mandible and segment the free fibular flap. The cutting guide should be fit the patient’s anatomy, easy to fix to the mandible and fibula bone and not affected the operation. Cutting guides and 3D model were manufactured in polyamide using a three-dimensional (3D) printer (SPS450, The national engineering research center of rapid manufacturing, Xi’an Jiaotong University, China) (Fig. 2). The model and guides were then sterilized for intraoperative use.

**Figure 3:**
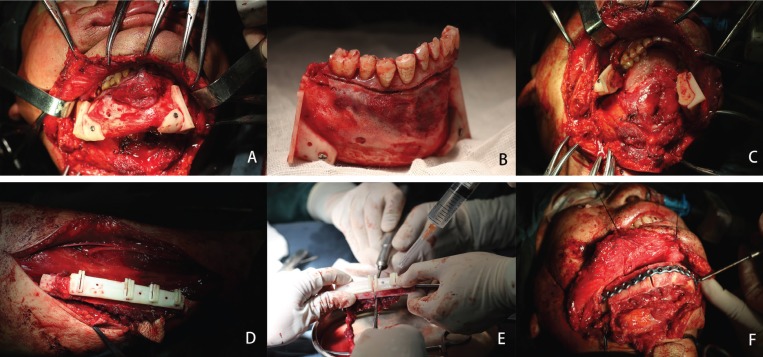
The surgical phase (A. Cutting guide was fixed on the mandible; B. Making osteotomies of mandible according to the guide; C. After resection of the mandible; D. Cutting guide was fixed on the fibula; E. Making osteotomies of fibula according to the guide; F. The proper fibular segments were transferred to reconstruct the defect).

The titanium reconstruction plate was pre-bent along the contours of the model to save operative time (Fig. 2). In the surgical phase, the sterilized cutting guide was temporarily fixed to the mandible using monocortical screws, and a reciprocating saw blade was inserted into slots of the cutting guide to make osteotomies. After resection of the mandible, the reconstruction plate was placed as the plan dictated, spanning the defect, and temporarily fixed with at least 2 screws on each side. The osteotomies of harvested fibula bone using the same protocol at the lengths and angles required to replicate the virtual plan (Fig. 3). The proper fibular segments were transferred to reconstruct the defect. Remaining holes were of plate drilled and screws were placed as planning. Vascular anastomosis and wound closure were performed using the standard method.

-Data analysis

In control group, mandibular osteotomies were performed on the basis of the appropriate margins observed on the CT scans. Titanium reconstruction plate was bent along the contours of the native mandible and was prefixed to the mandible before mandibular lesion resection, then was removed. When the resection was finished, the plate was fixed again and used to guide fibular osteotomies. Fibular osteotomies were performed according to the shape and length of the mandibular defects and were mostly dependent on the surgeon’s experience. Finally, fix the fibular segments to the mandible. In cases the native mandible was absent, the resection was performed, and the plate was bent in such a way as to best maintain centric occlusion of the remaining dentition. The total operative times and reconstructive times was recorded into groups. Reconstructive times start to count when the pedicle of the fibular flap was divided and stop to record when the vascular anastomosis was finished.

**Figure 4:**
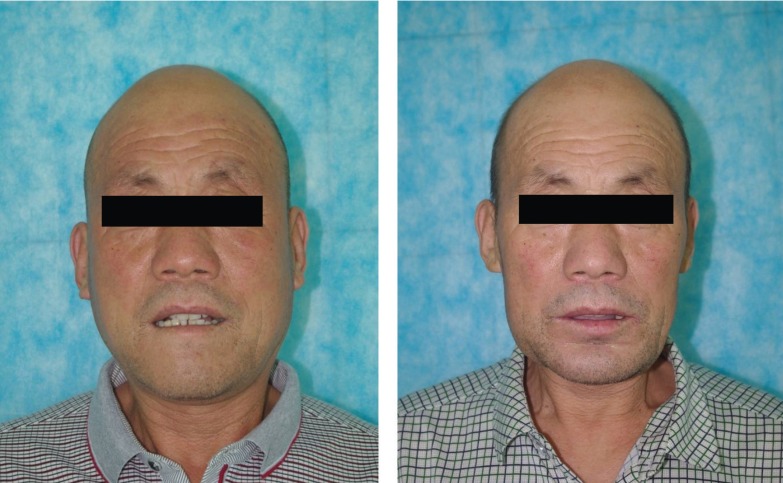
Preoperative and postoperative appearance of the patient (A. Preoperative appearance; B. Postoperative appearance).

The follow-up period was 1, 3, 6 months after the surgical procedure (Fig. 4). Postoperative occlusion and appearance satisfaction were evaluated in the sixth month after the surgery. A postoperative CT scan was obtained for each patient 6 months after the surgery.

To assess the he accuracy of the reconstruction, we superimpose the images of the pre-operation and post-operation which were obtained from reformatted CT scan data in Mimic 16.0. 5 mandibular bony landmarks, bilateral condyle, bilateral gonion, Gnathion, were used to compare the final results for the computer-assisted group and the control group. The measurement method described by Zhang (13) was used. Intercondylar distance, intergonial angle distance and anteroposterior distance (using a perpendicular line drawn from the mandibular midline to the center point of the intercondylar length) and gonial angle were measured.

Continuous data, reported as mean±standard deviation, were compared using the unpaired t test if the data were normally distributed, and the Mann–Whitney U test if they were not. Categorical data were compared using the chi square test or Fisher exact test, as appropriate. All tests were two-tailed. *P* values <.05 were accepted as significant.

## Results

This retrospective study involved 30 consecutive patients (19 male and 11 female patients), with an average age of 39.1 years (range, 21-63 years) who underwent surgical resection. Diagnoses included ameloblastoma, keratocystic odontogenic tumor (KCOT), ossifying fibroma and SCC of gingiva. In most patients, the primary tumor was an ameloblastoma (n=18,60%) ( Table 1).

**Table 1:**
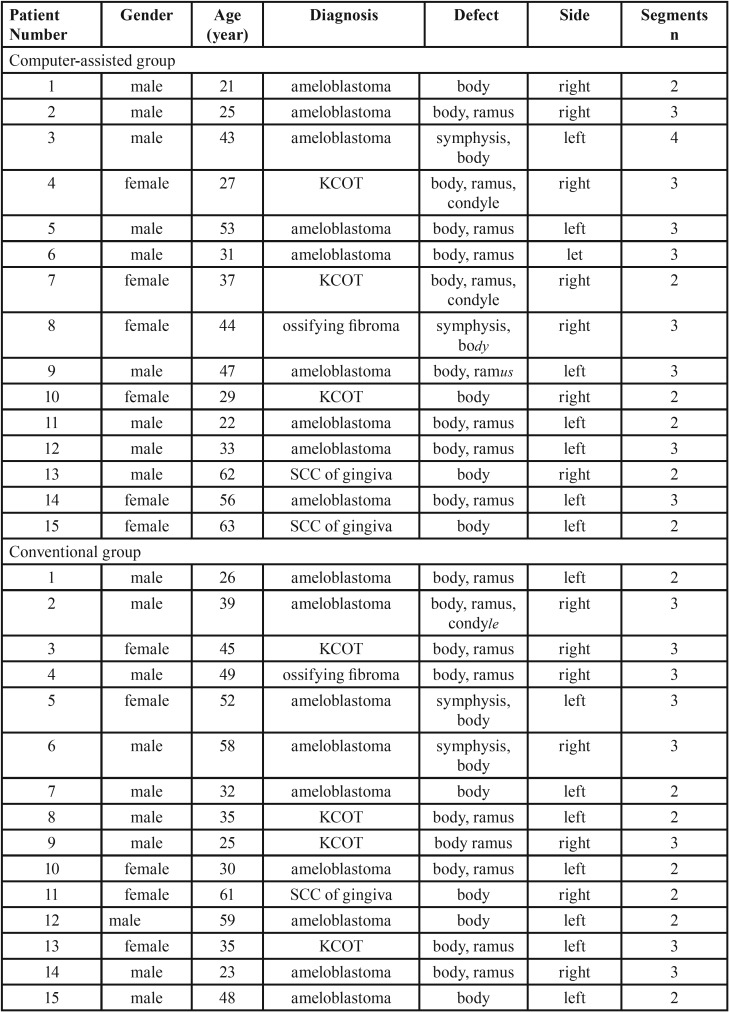
Patient demographic data.

All the bone flaps in two group were survived. The postoperative occlusion of the 30 patients after the surgical procedure was stable. All patients achieved a symmetric mandibular contour based on the clinical examination and were satisfied with the facial appearance. There were no wound-related complications.

The mean reconstructive times was 1.60±0.37 and 2.58±0.45 hours in the computer-assisted group and conventional group, respectively; The total operative times was 5.54±0.50 and 6.54±0.70 hours in the computer-assisted group and conventional group, respectively. The mean total operative times and reconstructive times was compare to the conventional group, both operative times and reconstructive times were shorter in computer-assisted group ( Table 2).

**Table 2:**
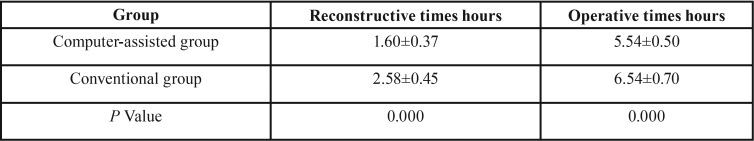
Compare the operative times between the CAD and 3D printing modeling aid group (Computer-assisted group) and Conventional group.

The mean difference between the preoperative and postoperative intercondylar distances in the computer-assisted and conventional group was 2.92±1.15 and 4.48±1.41mm, respectively; the mean difference between the preoperative and postoperative intergonial angle distances was 2.93±1.19 and 4.79±1.48mm, respectively; the mean difference between the preoperative and postoperative anteroposterior distances was 4.31±1.24 and 5.61±1.41mm, respectively; and the mean difference between the preoperative and postoperative gonial angles was 3.85±1.68° and 5.88±2.12°, respectively ( Table 3).

**Table 3:**
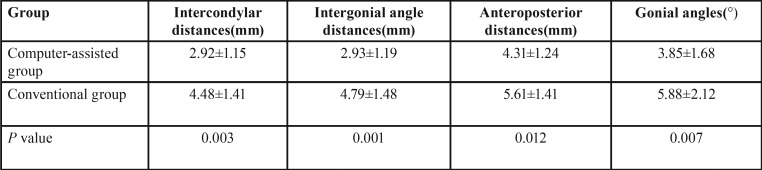
Mean Difference in Position of Bony Landmarks Between Preoperative and Postoperative Mandibles in two group.

## Discussion

The use of vascularized bone flaps has become the gold standard for mandibular reconstruction. The fibula flap is the workhorse flap for mandibular reconstruction due to its thickness, length, and bone uniformity, which make it the ideal support for implants and good match for the alveolar ridge (2,13-16). However, the greatest challenges that remains is how to most accurately shape vascularized bone flaps so that facial symmetry as well as function are best restored and minimize the operative time of such complex surgery in the same time. Conventional techniques base on the surgeons’ experience and lack effective quantitative strategies. Virtual surgical planning including CAD/CAM has changed the way of bony reconstruction in recent years. This technique gave improved results in terms of reduced operating time and good aesthetic and functional results (10,17,18).

In the present study, we used virtual planning and 3D printing modeling to assist with mandibular reconstruction and compared the outcomes with conventional method. Patient characteristics and defects were very similar in two groups. There was no significant difference in the complication observed between the two groups, suggesting that use of this technique is not associated with any potential hazards to patient safety. This is similar to the previously reported (9). However, the computer-aided preoperative planning and 3D printing modeling help the surgeon to reduce the operative times. Seruya *et al.* reported significantly decreased flap ischemia time, from 170 to 120 minutes in a series of 10 computer-assisted mandibular reconstructions (19). Zhang *et al.* reported virtual surgical planning decreased the duration of ischemia compared to the conventional group (13). This was also observed in several other studies (10,20). In present study, results show that both operative times and reconstructive times were shorter in computer-assisted group compared with the conventional group. The decrease in surgical time, especially duration of ischemia, implies fewer postoperative complications.

Another potential benefit of computer-assisted surgery is the improvement of accuracy of mandibular reconstruction (20). The cutting guides used for mandibular and fibular osteotomies and pre-bent titanium plates provided a faithful duplication of the preoperative virtual plan which allowed the surgeons to assemble the fibular segments into the defect of mandible as preoperative design and minimize the adjustments for the final inset compared to conventional method. Although using varied measurement methods, several studies have reported the virtual surgical plan could improve the accuracy of mandibular reconstructio(10,12,13,17,21). We used the measurement described by Zhang (13) in this study. Results show that the mean differences between the preoperative and postoperative intercondylar distances, intergonial angle distances, anteroposterior distances, and gonial angles were smaller in the computer-assisted group compared with conventional group, which indicated that the accuracy of the computer-assisted group was greater than that of conventional group. Surgical navigation is a useful tool that can verify the actual position with preoperative virtual plan during surgery (22). Yao Yu *et al.* fund that combined application of the CAD and surgical navigation resulted in a more accurate outcome for mandibular reconstruction with free fibula flap (4). However, time consumption, learning curve and costs should be taken into account when surgical navigation is used as a surgical tool (23).

**Figure 5:**
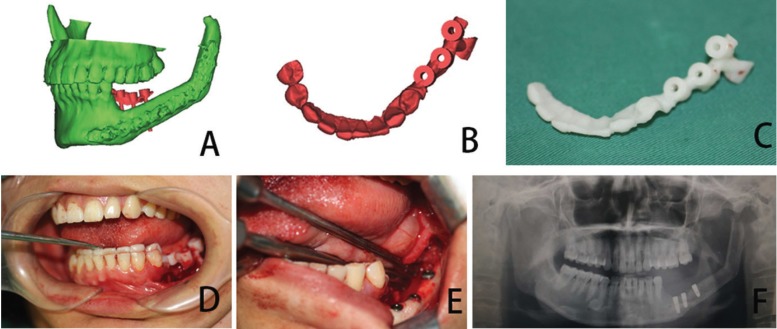
Computer assisted oral rehabilitation with implant in the fibular transplants. (A. Simulation of implant surgery; B. Implant guide design; C. Making the guide using 3D printer; D. The guide was in position according to the teeth. E. Performing the implant surgery according to the guide; F. Postoperative panoramic radiograph).

Base on the fibular transplants, using dental implants for oral rehabilitation has been frequently used following reconstruction of the mandible and has proven to be a reliable method (24). However, the position of the dental implant was difficult during surgery compared to the conventional implant. We also use virtual planning and 3D printing modeling for the tooth implant in the fibular transplants (Fig. 5). The process is similar. With help the guide plate, the implants are positioned exactly where planned virtually. The reconstructed mandible must be properly aligned with the maxilla in both the horizontal and vertical planes, with adequate intermaxillary space to insert a prosthesis (25,26). Therefore, we recommend the position of implant should be considered in the virtual plan before the mandibular reconstruction, whether taking the dental implant in the first-stage or second-stage.

Although the virtual planning and 3D printing modeling have such advantages in mandibular reconstruction. We also should realize the limitation of this method. An important limitation we encountered was the potential for the extent of resection to change during the operation. It may change the location of osteotomies and reduce the usefulness of this technique, Because the bone segment length or number of osteotomies should adjust the change and the pre-bent titanium plate may doesn’t work. This kind of situation are more likely to happen in advanced malignant tumor and osteoradionecrosis (27,28). Another factor which was not programmed into the computer algorithm was soft tissue mask, which also affect the final outcomes and patient satisfaction of mandibular reconstruction (29). In our experience, this technique should apply on the selected patients at present, such as mainly bone defect and easy to determine the surgical margin prior to surgery. In this study, we select the benign tumor of mandible or early stage of SCC of gingival and consider such conditions was the excellent indication for computer-aid mandibular reconstruction. It also may be useful in patients with missing mandibular segments or second-stage reconstruction. In addition, close communication with the resecting surgeon contribute to minimize changes from initial virtual plan.

The findings of this study indicate that the use of computer-assisted design and 3D printing modeling in selected patients could saving operation time and improve the accuracy of mandible reconstruction. We believe that this technology for mandibular reconstruction will become a used method and improve patients’ quality of life.
